# Interleukin-1β rs1143627 polymorphism with susceptibility to periodontal disease

**DOI:** 10.18632/oncotarget.15612

**Published:** 2017-02-22

**Authors:** Wei Huang, Bing-Yang He, Jun Shao, Xiao-Wei Jia, Ya-Di Yuan

**Affiliations:** ^1^ Department of Stomatology, Zhuhai People's Hospital, Zhuhai Hospital Affiliated With Jinan University, Zhuhai 519099, Guangdong Province, China; ^2^ Department of Stomatology, The First Affiliated Hospital of Zhengzhou University, Zhengzhou 450052, Henan Province, China; ^3^ Department of Stomatology, Guangzhou Hospital of Integrated Traditional and West Medicine, Guangzhou 510800, Guangdong Province, China

**Keywords:** interleukin-1, periodontitis, periodontal disease, meta-analysis, polymorphism

## Abstract

Association between interleukin-1 beta (*IL-1β*) rs1143627 polymorphism and periodontal disease susceptibility was inconsistent; hence we performed this meta-analysis to explore the precise correlation between them. The degree of association was appraised through calculating pooled odds ratio (OR) and its 95% confidence interval (CI). The databases known as PubMed, Embase, and Chinese National Knowledge Infrastructure were searched up to October 26, 2016. A total of 8 eligible case-control studies were finally included, which involved 229 aggressive periodontitis patients, 382 chronic periodontitis patients, and 555 healthy controls. All the five genetic models revealed a non-significant association between *IL-1β* rs1143627 polymorphism and periodontal disease susceptibility (TT vs. CC: OR = 1.22, 95% CI = 0.80-1.87; CT+TT vs. CC: OR = 0.66, 95% CI = 0.44-1.01; TT vs. CT + CC: OR = 1.19, 95% CI = 0.81-1.74; T vs. C: OR = 0.92, 95% CI = 0.81-1.12; CT vs. CC: OR = 0.92, 95% CI = 0.69-1.23). Sensitivity analyses indicated that the results were robust and the subgroup analyses reached similar conclusions. *IL-1β* rs1143627 polymorphism is not related to periodontal disease susceptibility in the overall population based on the current evidence, but further studies are required in more large scale sample size with risk factor adjusted.

## INTRODUCTION

Periodontal disease, a multifactorial disease, is mainly composed of chronic periodontitis (CP) and aggressive periodontitis (AgP) [1–3]. Periodontal disease, which is a risk factor of systemic diseases, such as head and neck cancer [4], diabetes [5], cardiovascular disease [6–7], erectile dysfunction [8], preterm birth, and low birthweight [9] has become a major public health problem. Therefore, it is of great significant to detect periodontal disease activity and to predict treatment efficacy genetically. A variety of publications have reported the potential association between interleukin (*IL*) polymorphisms and periodontal disease. *IL-1β* (beta) is highly polymorphic, and three polymorphisms that lead to transitions between C and T at positions -31(T→C, rs1143627), -511(C→T, rs16944), and +3954/3953(C→T, rs1143634) base pairs (bp) from the transcriptional site have been widely researched [1, 10–11]. Meta-analyses have proven that *IL-1β* rs1143634 polymorphism is significantly connected with the increased risk of CP [12–13], especially for white adults [14]; whereas, no significant association was discovered between *IL-1β* rs16944 polymorphism and CP susceptibility [15]. In addition, *IL-1β* rs1143634 polymorphism is correlated with CP [12–14] but has nothing to do with AgP [1].

An increasing number of studies have examined the association between *IL-1β* rs1143627 polymorphism and periodontal disease, but as far as we know, no meta-analyses have been carried out about this issue yet. Therefore, the questions of whether *IL-1β* rs1143627 polymorphism is related to periodontal disease or not, and whether there is a difference of such relationship between CP and AgP still remain uncertain. As a consequence, we conducted this meta-analysis in accordance with the Preferred Reporting Items for Systematic Reviews and Meta-Analyses (PRISMA) statement [16] in order to answer those questions.

## RESULTS

### Study identification and characteristics

**Figure 1:**
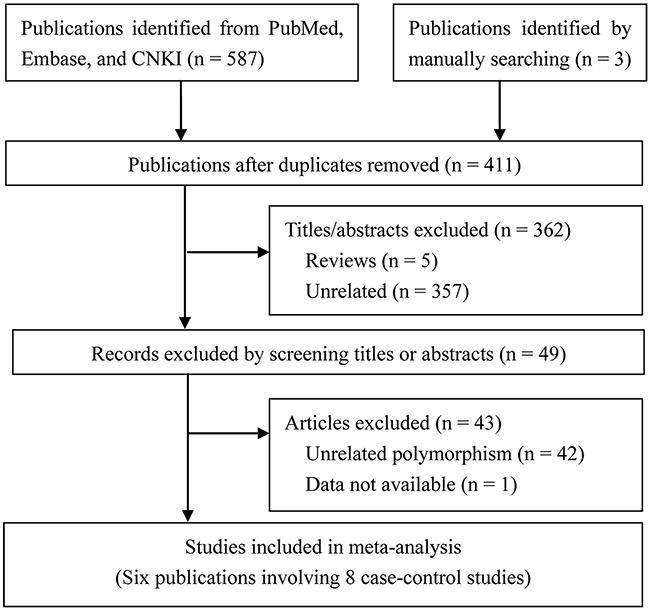
Study selection flow diagram

The initial search identified 350 publications and 6 eligible publications involving 8 case-control studies [17–22] were finally included. The literature search and selection process are shown in Figure 1.

The characteristics and relevant data of all included studies are shown in Table 1. All studies were case-control studies, totally containing 229 AgP patients, 382 CP patients, and 555 healthy controls. Two studies included both AgP and CP [19–20], which were treated as 4 independently case-control studies; while 3 case-control studies [17–18, 22] focused on CP patients and only 1 [19] focused on AgP patients. Among these studies, 5 were carried out in Asians, and 2 were performed in Jordanian, while only 1 was conducted in Italians and. All the studies conformed to HWE (*p* > 0.05).

**Table 1:** Characteristics of included studies in meta-analysis

Study	Country (Ethinicity)	Type	Case/Control					Genotyping method	HWE	Smoking status
			Sample	Mean age	Male:Female	C allele	T allele			
Komatsu 2008	Japan (Asian)	CP	113/108	57.4±0.9/47.4±1.7	43:70/47:61	107/108	119/108	NA	Yes	None
Scapoli 2010	Italy (unrelated Caucasian Italians)	AgP	95/121	43.4 ±7.7/30.1 ±4.7	29:66/60:61	1.002 (0.645-1.558)*		MassARRAY	Yes	NA
Kobayashi 2009	Japan (Asian)	CP	117/108	51.9±0.9/51.2±1.2	86:31/75:35	120/104	114/112	TaqMan	Yes	Mixed
Shete 2010	India (Asian)	CP	43/107	35.21/NA	7:36/NA	61/147	25/67	PCR-RFLP	Yes	None
Shete 2010	India (Asian)	AgP	54/107	25.9/NA	20:34/NA	76/107	32/67	PCR-RFLP	Yes	None
Karasneh 2011	Jordan (Jordanian)	CP	100/80	40.43±11.25/22.28±5.43	56:44/48:32	117/80	83/104	PCR-RFLP	Yes	Mixed
Karasneh 2011	Jordan (Jordanian)	AgP	80/80	29.15±7.92/22.28±5.43	22:58/48:32	100/80	60/104	PCR-RFLP	Yes	Mixed
Amirisetty 2014	India (Asian)	CP	29/31	41.3/41.8	23:6/23:8	36/31	36/22	PCR-RFLP	Yes	None

Notes: CP, chronic periodontitis; AgP, aggressive periodontitis; NA, not available; HWE, Hardy-Weinberg equilibrium; None, non-smokers; Mixed, with both ever smokers and non-smokers; *, odds ratio with its 95% confidence interval.

### Meta-analysis

Table 2 shows the results of heterogeneity test, subgroup analyses, and overall estimates.

**Table 2:** Overall and subgroup analyses of meta-analysis

Genetic comparison	Overall and subgroup analysis	No. of studies	Heterogeneity		Model	Meta-analysis	
			p	*I*^2^(%)		OR(95%CI)	p
T vs. C	Overall	8	0.78	0.00	Fixed	0.92(0.81-1.12)	0.57
	AgP	3	0.52	0.00	Fixed	0.92(0.71-1.20)	0.55
	CP	5	0.63	0.00	Fixed	0.97(0.80-1.19)	0.79
	Asian	5	0.68	0.00	Fixed	0.97(0.80-1.16)	0.71
	Jordanian	2	0.31	3.82	Fixed	0.85(0.59-1.23)	0.40
	Italian	1	1.00	0.00	Fixed	1.11(0.64-1.92)	0.70
	Mixed	3	0.34	8.52	Fixed	0.98(0.76-1.28)	0.90
	None	4	0.72	0.00	Fixed	0.91(0.74-1.13)	0.42
TT vs. CC	Overall	6	0.33	13.32	Fixed	1.22(0.80-1.87)	0.36
	AgP	2	0.50	0.00	Fixed	1.74(0.88-3.45)	0.11
	CP	4	0.31	17.10	Fixed	0.98(0.57-1.68)	0.93
	Asian	4	0.72	0.00	Fixed	0.84(0.48-1.45)	0.52
	Jordanian	2	0.95	0.00	Fixed	2.10(1.09-4.07)	0.03
	Mixed	3	0.15	47.51	Fixed	1.39(0.83-2.31)	0.21
	None	3	0.55	0.00	Fixed	0.92(0.43-1.97)	0.84
CT vs. CC	Overall	6	0.80	0.00	Fixed	0.92(0.69-1.23)	0.58
	AgP	2	0.33	0.00	Fixed	0.74(0.46-1.20)	0.22
	CP	4	0.98	0.00	Fixed	1.05(0.72-1.52)	0.80
	Asian	4	0.56	0.00	Fixed	0.87(0.60-1.28)	0.49
	Jordanian	2	0.72	0.00	Fixed	0.99(0.63-1.55)	0.96
	Mixed	3	0.93	0.00	Fixed	1.00(0.69-1.45)	1.00
	None	3	0.43	0.00	Fixed	0.81(0.50-1.29)	0.37
TT vs. (CC+CT)	Overall	6	0.15	38.91	Fixed	1.19(0.81-1.74)	0.37
	AgP	2	0.67	0.00	Fixed	1.98(1.04-3.80)	0.04
	CP	4	0.22	32.14	Fixed	0.92(0.57-1.46)	0.71
	Asian	4	0.41	0.00	Fixed	0.84(0.52-1.36)	0.49
	Jordanian	2	0.84	0.00	Fixed	2.11(1.14-3.91)	0.02
	Mixed	3	0.06	63.53	Random	1.40(0.66-2.99)	0.38
	None	3	0.29	18.41	Fixed	1.02(0.49-2.10)	0.96
(TT+CT) vs. CC	Overall	6	0.04	57.80	Random	0.66(0.44-1.01)	0.06
	AgP	2	0.27	18.76	Fixed	0.45(0.30-0.68)	<0.01
	CP	4	0.16	41.19	Fixed	0.86(0.60-1.22)	0.39
	Asian	4	0.09	54.35	Random	0.59(0.34-1.01)	0.05
	Jordanian	2	0.05	72.43	Random	0.82(0.37-1.81)	0.62
	Mixed	3	0.14	49.45	Fixed	0.83(0.59-1.18)	0.30
	None	3	0.21	35.43	Fixed	0.45(0.29-0.69)	<0.01

Notes: AgP, aggressive periodontitis; CP, chronic periodontitis; OR, odds ratio; CI, confidence interval; Mixed, with both non-smokers and ever smokers; None, non-smokers.

**Figure 2:**
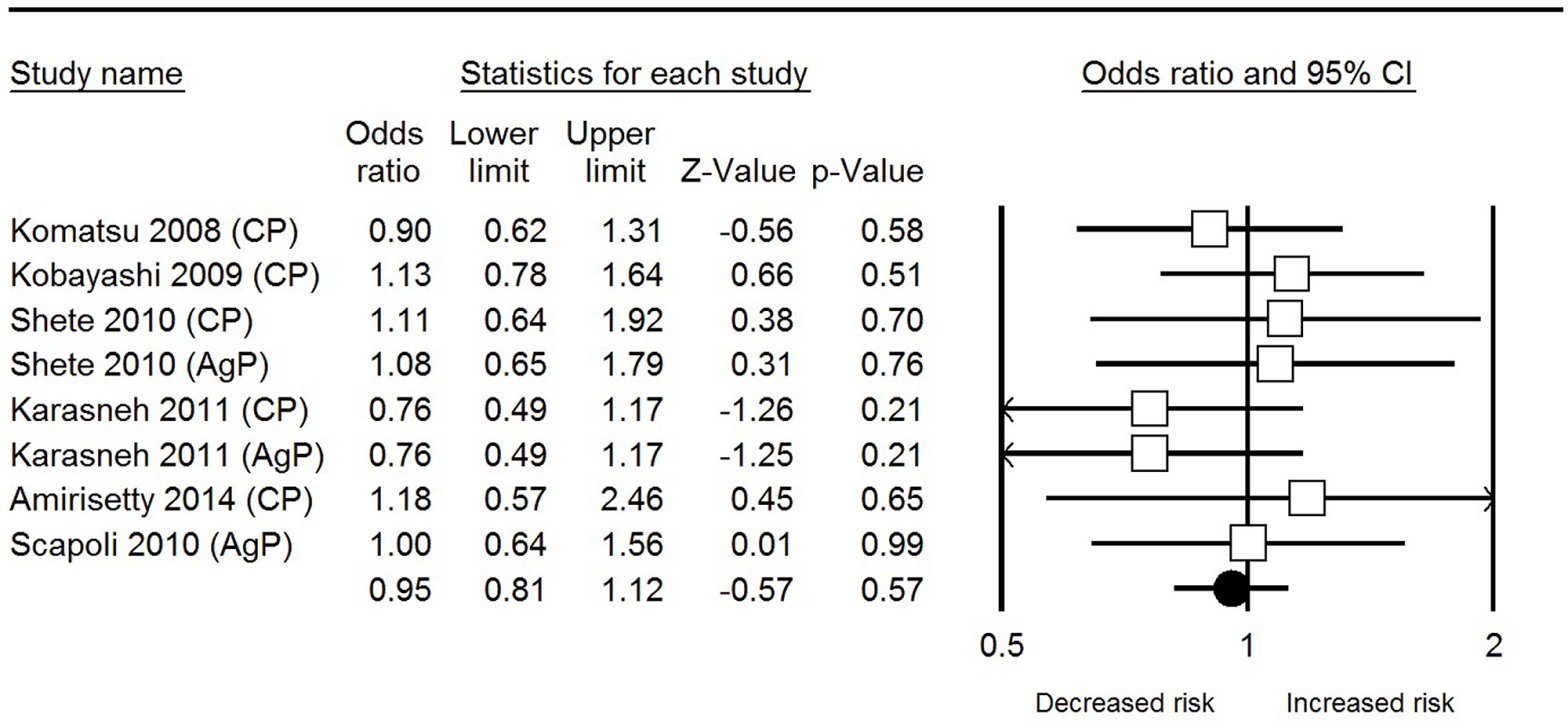
Forest plot of IL-1β rs1143627 polymorphism and periodontal disease in allele comparison

**Figure 3:**
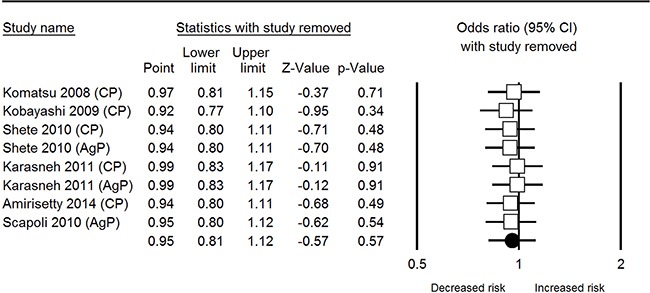
Sensitivity analysis plot of IL-1β rs1143627 polymorphism and periodontal disease in allele comparison

In total analysis, non-significant correlation was observed between *IL-1β* rs1143627 polymorphism and periodontal disease susceptibility under all 5 genetic models [T vs. C: odds ratio (OR) = 0.92, 95% confidence interval (CI) = 0.81-1.12, *I*^2^= 0% (Figure 2); TT vs. CC: OR = 1.22, 95% CI = 0.80-1.87, *I*^2^= 13.32%; CT vs. CC: OR = 0.92, 95% CI = 0.69-1.23, *I*^2^= 0%; CT+TT vs. CC: OR = 0.66, 95% CI = 0.44-1.01, *I*^2^= 57.8%; TT vs. CT + CC: OR = 1.19, 95% CI = 0.81-1.74, *I*^2^= 38.91%]. Sensitivity analyses of each genetic model all revealed robust results (Figure 3).

Subgroup analysis based on smoking status also revealed non-significant association. However, after stratified analysis by ethnicity, TT vs. CC (OR = 2.10, 95%CI = 1.09-4.07) and TT vs. CT + CC (OR = 2.11, 95%CI = 1.14-3.91) stated that *IL-1β* rs1143627 polymorphism was associated with the increased risk of PD in Jordanian population; meanwhile, in stratified analysis by periodontal disease type, the TT vs. CT + CC (OR = 1.98, 95%CI = 1.04-3.80) showed that *IL-1β* rs1143627 polymorphism was connected with the increased risk of AgP while the CT+TT vs. CC (OR = 0.45, 95%CI = 0.30-0.68) revealed a decreased risk of AgP.

### Publication bias

**Figure 4:**
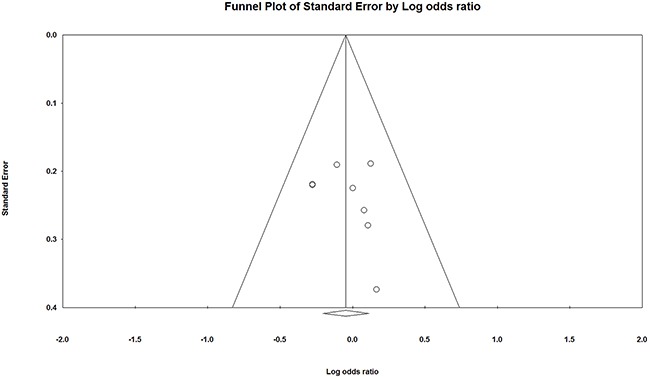
Funnel plot of IL-1β rs1143627 polymorphism and periodontal disease in allele comparison

There was no obvious asymmetry in the funnel plot through visual inspection (Figure 4). The Egger's test of all 5 genetic models also showed no evidence of significant publication bias (T vs. C: *p* = 0.46; TT vs. CC: *p* = 0.44; CT vs. CC: *p* = 0.76; (TT+CT) vs. CC: *p* = 0.70; TT vs. (CT + CC): *p* = 0.81).

## DISCUSSION

Our meta-analysis, based on 8 eligible case-control studies, demonstrated that there was no obvious association of *IL-1β* rs1143627 polymorphism with periodontal disease susceptibility in total analysis. The sensitivity analyses supported that the overall analyses were robust, and the majority of subgroup analyses concluded similar results in the overall populations. Smoking, which is a classical risk factor of periodontal disease [23] influences the prognosis of periodontal disease [24]. Hence, we performed a subgroup analysis according to the smoking status of participants. The results showed non-significant relation of periodontal disease risk with either non-smokers or mixed smokers (including ever smokers and non-smokers). Due to the lack of sufficient data, we could not detect whether *IL-1β* rs1143627 polymorphism is a co-factor of smoking in periodontal disease. Further studies should divide the populations into smokers and non-smokers, or only contain smokers to provide evidences for the collective effect of smoking with this polymorphism.

The current study was the first meta-analysis designed to investigate the correlation of IL-1β rs1143627 polymorphism with the risk of periodontal disease, and revealed a negative result, showing the lack of significant association. The results could be explained by some clues from published meta-analyses. First, the different polymorphisms in the same IL gene could show different results. For example, in 2009, Shao et al [25] conducted a systematic review and meta-analysis which indicated that only IL-6 C572G polymorphism was associated with periodontitis. Then a meta-analysis by Zhong et al [26] in 2012 illustrated that IL-10 C-819T and C-592A polymorphisms were in connection with CP while A-1082G polymorphism was not. Second, the association of the same gene polymorphism may be different in CP and AgP. For example, IL-1α C-899 (+4845)T polymorphism was related to the increased risk of CP [27] but not associated with AgP [28]. Since there were few studies included in our analysis, the current results should be treated with caution because the results may change along with alterations in studies populations, disease type or other relevant factors.

The major limitations of our meta-analysis were the small sample size and limited number of included studies. The results also changed as sample size increased. In 2008, Nikolopoulos et al [13] conducted a meta-analysis based on 10 case-control studies which informed no association of IL-1β rs1143634 polymorphism with CP in Caucasian populations. Yet, the later meta-analysis based on 16 studies by Deng et al [12] in 2013 put forward that IL-1β rs1143634 polymorphism was associated with increased risk of CP in Caucasian populations. In consequence, further studies are necessary to verify which conclusion is accurate. Moreover, we only enrolled studies published in English or Chinese language, suggesting that the publications in other languages were neglected. Furthermore, although the heterogeneity apart from contrast TT+CT vs. CC was acceptable in 4 genetic models, we still could not ignore the potential influences of heterogeneity. We failed to find an explanation for the heterogeneity of model TT+CT vs. CC being the largest. Meta-analysis was a secondary and observational study [29–30], thus it was limited by the quality of primary studies. Although we had conducted a more comprehensive search, the complete elimination of limitation still could not be guaranteed. The effects of gene - gene or gene - environment interactions were not analyzed owing to the lack of original data. In other words, our results were based on unadjusted data and these limitations might influence our final conclusions.

In summary, our meta-analysis suggests that *IL-1β* rs1143627 polymorphism is not associated with periodontal disease on the basis of current available evidence. However, we suggest that the results should be examined in further better-designed researches with larger sample size and multiple ethnic groups considering the above mentioned limitations, in order to measure the relationship of *IL-1β* rs1143627 polymorphism with periodontal disease development more comprehensively and precisely.

## MATERIALS AND METHODS

### Eligible criteria

Studies would be included if they met all the following criteria: (1) patients were clearly diagnosed with periodontal disease (either CP, AgP, or both) and the controls were periodontally healthy people without periodontitis; (2) studies explored the association between IL-1β rs1143627 polymorphism and periodontal disease susceptibility using a case-control or cohort study design; (3) full data on OR and 95% CI were reported in each group, or sufficient data were provided to calculate them; and (4) if two or more publications were from the same institute, we would compared their detailed basic information and then chose the more comprehensive one.

### Search strategy

Two authors independently searched multiple online electronic databases known as PubMed, Embase, and Chinese National Knowledge Infrastructure (CNKI) up to October 26, 2016. The following key words were used in search strategy: “*IL-*1” or “interleukin-1”, “periodontal diseases” or “periodontitis”, and “polymorphism” or “mutation” or “variant” or “haplotype”. Moreover, all listed references of eligible studies and recently reviews were also screened for purpose of identifying the additional relevant publications. Only the studies published in Chinese or English were selected.

### Data extraction

Two authors independently collected and recorded the main information from each included case-control studies. Any discrepancies were resolved through discussion to come to a consensus. The extracted data were listed as follows: surname of the first author, year of publication, study design, country and ethnicity of included population, demographics, smoking status, number of cases and controls, genotype distributions, source of controls, genotyping method, and Hardy-Weinberg equilibrium (HWE) for controls. If HWE was not reported, we calculated the data according to genotype distributions of controls.

### Data analysis

Pooled OR and 95% CI were employed to estimate the association of *IL-1β* rs1143627 polymorphism with periodontal disease risk was evaluated under the allele comparison (T vs. C), homozygote comparison (TT vs. CC), heterozygote comparison (CT vs. CC), dominant model (TT+CT vs. CC), and recessive model (TT vs. CT + CC) [14–15, 31–35]. The between-study heterogeneity was assessed using the Cochrane Collaboration's *Q* statistic and *I*^2^ statistic [16, 36]. The fixed-effect model was applied while *p* > 0.1 in *Q* statistic and *I*^2^ < 50% in *I*^2^ statistic which suggested an acceptable heterogeneity; otherwise, the random-effects model was used. The sensitivity analysis was also performed by omitting each included study in turn for investigating the robustness of overall results [15, 31]. Besides, we carry out subgroup analyses to further evaluate the specific correlation between periodontal disease risk and *IL-1β* rs1143627 variant according to ethnicity, HWE status, periodontal disease type, and smoking status if it was appropriate. Publication bias was assessed with the method of funnel plot and Egger's test [37]. All the statistical analyses were carried out using the Comprehensive Meta-Analysis v2 software [15, 31, 38].
